# An audit to look at the prescribing of psychotropic medication in the general adult inpatient setting in patients with emotionally unstable personality disorder

**DOI:** 10.1192/bjo.2021.136

**Published:** 2021-06-18

**Authors:** Declan Hyland, Charlie Daniels, Iulian Ionescu, Christina Houghton, Katie Goodier, Simon Graham

**Affiliations:** 1 Core Trainee 2 in Psychiatry, Spring House, Liverpool, Mersey Care NHS Foundation Trust; 2 Consultant Psychiatrist, Clock View Hospital, Liverpool, Mersey Care NHS Foundation Trust; 3 Higher Trainee in Medical Psychotherapy, Spring House, Liverpool, Mersey Care NHS Foundation Trust; 4 Foundation Year 2 trainee, Clock View Hospital, Liverpool, Mersey Care NHS Foundation Trust; 5 Foundation Year 1 Trainee, Clock View Hospital, Liverpool, Mersey Care NHS Foundation Trust; 6 Consultant in Medical Psychotherapy, Spring House, Liverpool, Mersey Care NHS Foundation Trust

## Abstract

**Aims:**

To assess the frequency of prescription of psychotropic medication in patients with a primary diagnosis of emotionally unstable personality disorder (EUPD) following admission to Clock View Hospital, an inpatient unit in Mersey Care NHS Foundation Trust.

**Method:**

A retrospective analysis of the electronic (RiO) record of 50 patients discharged from Clock View Hospital between 1 January 2020 and 1 November 2020 was performed to assess prescribing practice.

Twenty-five patients with a diagnosis of EUPD and no associated psychiatric comorbidities were included in the sample, as well as 25 patients with a diagnosis of EUPD and associated psychiatric comorbidities.

**Result:**

80% of the 25 patients with EUPD and associated psychiatric comorbidities were prescribed psychotropic medication prior to admission to hospital (56% an antidepressant, 24% a mood stabiliser, 60% an antipsychotic and 8% a benzodiazepine). 64% of patients were prescribed two or more psychotropic medications. 28% were initiated on new psychotropic medications following admission. For four of the seven prescriptions commenced on psychotropic medication, prescribing practice was as advised in Mersey Care's EUPD guidelines.

Of the 25 patients with EUPD and no associated psychiatric comorbidities, 96% of the patients were prescribed psychotropic medication prior to admission to hospital (56% an antidepressant, 20% a mood stabiliser, 72% an antipsychotic and 12% a benzodiazepine). 68% of patients were prescribed two or more psychotropic medications. Following admission, 28% of patients were initiated on new regular psychotropic medications. For five of the eight prescriptions for new psychotropic medication, prescribing practice was as advised in Mersey Care's EUPD guidelines.

78% of the 50 patients were prescribed as required (PRN) psychotropic medication. In 21 patients, PRN medication was prescribed for longer than one week.

**Conclusion:**

There is a higher rate of prescribing of antipsychotic prescription in those EUPD patients with no psychiatric comorbidities compared to associated psychiatric comorbidities (72% vs 60%). Surprisingly, there was a lower rate of psychotropic polypharmacy in those with psychiatric comorbidities.

Use of PRN psychotropic medication for longer than a week was higher in those patients with psychiatric comorbidities compared to those without psychiatric comorbidities (58% vs 50%). Benzodiazepines were overwhelmingly the most consistently prescribed PRN medication for patients with EUPD.

One action to consider would be highlighting the importance of trialling psychologically-minded interventions and supportive psychotherapy prior to initiation of psychotropic medication. There also needs to be consideration to use of the sedative antihistamine promethazine as a first-line PRN medication for acute agitation.

